# Ligase-mediated synthesis of Cu^II^-responsive allosteric DNAzyme with bifacial 5-carboxyuracil nucleobases†

**DOI:** 10.1039/d3sc05042d

**Published:** 2024-01-20

**Authors:** Yusuke Takezawa, Hanci Zhang, Keita Mori, Lingyun Hu, Mitsuhiko Shionoya

**Affiliations:** a Department of Chemistry, Graduate School of Science, The University of Tokyo 7-3-1 Hongo, Bunkyo-ku Tokyo 113-0033 Japan takezawa@chem.s.u-tokyo.ac.jp shionoya@chem.s.u-tokyo.ac.jp

## Abstract

A Cu^II^-responsive allosteric DNAzyme has been developed by introducing bifacial 5-carboxyuracil (caU) nucleobases, which form both hydrogen-bonded caU–A and metal-mediated caU–Cu^II^–caU base pairs. The base sequence was logically designed based on a known RNA-cleaving DNAzyme so that the caU-modified DNAzyme (caU-DNAzyme) can form a catalytically inactive structure containing three caU–A base pairs and an active form with three caU–Cu^II^–caU pairs. The caU-DNAzyme was synthesized by joining short caU-containing fragments with a standard DNA ligase. The activity of caU-DNAzyme was suppressed without Cu^II^, but enhanced 21-fold with the addition of Cu^II^. Furthermore, the DNAzyme activity was turned on and off during the reaction by the addition and removal of Cu^II^ ions. Both ligase-mediated synthesis and Cu^II^-dependent allosteric regulation were achieved by the bifacial base pairing properties of caU. This study provides a new strategy for designing stimuli-responsive DNA molecular systems.

## Abstract 2

A Cu^II^-responsive allosteric DNAzyme was designed by introducing bifacial 5-carboxyuracil (caU) bases that form both hydrogen-bonded caU–A and metal-mediated caU–Cu^II^–caU base pairs, and was synthesized by joining short caU oligomers with a DNA ligase.

## Graphical abstract

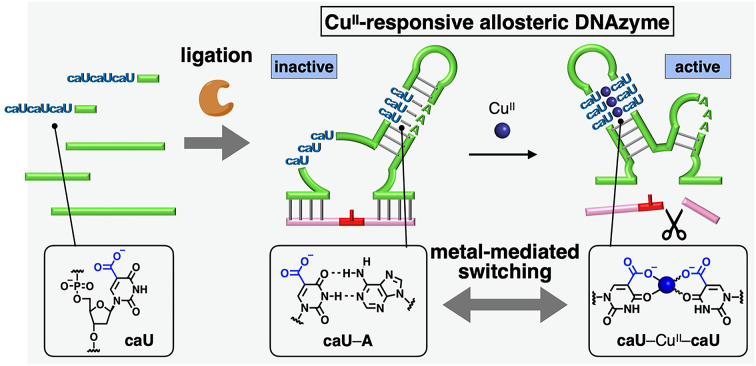

## Introduction

The highly sophisticated molecular recognition abilities of nucleic acids based on complementary hydrogen-bonded base pairing have led to a dramatic growth in the research field recognized as DNA nanotechnology.^1^ Numerous DNA nanodevices, sensors, and molecular machines have been created by controlling DNA hybridization and structures in response to stimuli such as DNA/RNA binding, pH changes, and light irradiation.^2^ Metal ions also serve as external stimuli to regulate the DNA structure and function, particularly by exploiting metal-mediated unnatural base pairing.^3^ Metal-mediated artificial base pairs are formed between two opposing ligand-type nucleobase analogs by complexation with a bridging metal ion. Metal-mediated base pairing generally stabilizes DNA duplexes, thus controlling DNA hybridization in a metal-dependent manner.

Aiming to switch DNA functions efficiently by metal complexation, we have recently established a new concept of metal-mediated base pair switching of bifacial 5-modified pyrimidine nucleobases.^4–7^ Bifacial bases such as 5-hydroxyuracil (U^OH^)^4,5^ and 5-carboxyuracil (caU)^6^ are designed to form metal-mediated self-base pairs (*e.g.*, U^OH^–Gd^III^–U^OH^) in the presence of certain metal ions and to form Watson–Crick-like base pairs with a natural nucleobase in DNA duplexes (*e.g.*, U^OH^–A). Based on the switching between U^OH^–A and U^OH^–Gd^III^–U^OH^ base pairs, Gd^III^-triggered DNA strand displacement reactions were demonstrated and Gd^III^-mediated control of DNA tweezer structures and DNAzyme functions was successfully achieved.^5^

**Fig. 1:**
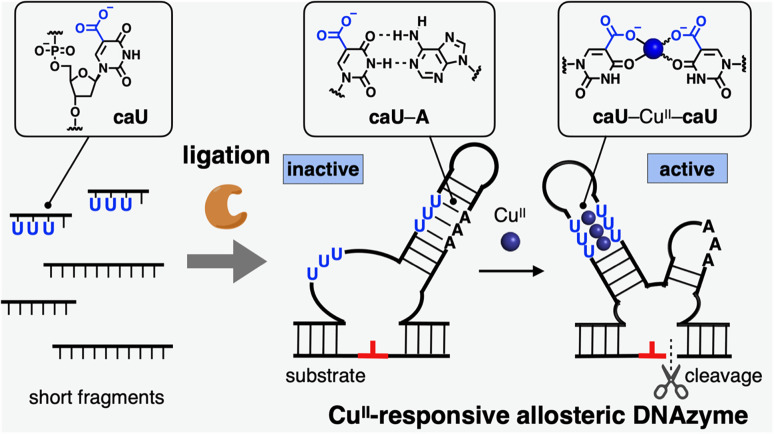
Ligase-mediated synthesis of a Cu^II^-responsive DNAzyme containing bifacial 5-carboxyuracil (caU) bases, which form hydrogen-bonded caU–A and metal-mediated caU–Cu^II^–caU base pairs. U in the figure represents a caU base.

In this study, a metal-responsive DNAzyme was newly developed by utilizing bifacial 5-carboxyuracil (caU) bases as metal binding sites (Fig. 1). DNAzymes are DNA molecules with catalytic activity that have been widely applied for the development of DNA-based biosensors and molecular machines.^8^ Of particular interest is the rational design of allosteric DNAzymes whose activity can be controlled in response to specific stimuli. Such stimuli-responsive DNAzymes are versatile components for building up deformable DNA nanoarchitectures as well as DNA reaction networks. Metal-responsive DNAzymes have been developed previously by incorporating metal-mediated base pairs such as Cu^II^-mediated hydroxypyridone base pairs (H–Cu^II^–H).^9^ The bifacial caU bases used in this study not only form hydrogen-bonded caU–A base pairs, but also Cu^II^-mediated caU–Cu^II^–caU base pairs with high metal selectivity.^6^ The caU–Cu^II^–caU base pairing significantly stabilizes the DNA duplexes (Δ*T*_m_ = +30.7 °C for a duplex with three caU–Cu^II^–caU pairs), whereas the caU–A base pairs are destabilized by the addition of Cu^II^ ions (Δ*T*_m_ = −7.3 °C for a duplex with three caU–A pairs by using 6 equiv. of Cu^II^ ions). Therefore, carefully designed DNAzymes containing caU bases were expected to exhibit good responsiveness to Cu^II^ ions. To prepare DNAzyme strands containing multiple caU nucleotides, enzymatic synthesis using a DNA ligase was also investigated. A standard T4 DNA ligase was utilized because it was reported to tolerate modified base pairs and backbones.^9*b*,*d*,10^ Thus, we expected that caU-containing short fragments could be enzymatically ligated to give long DNA strands modified with caU bases.

## Results and discussion

**Fig. 2:**
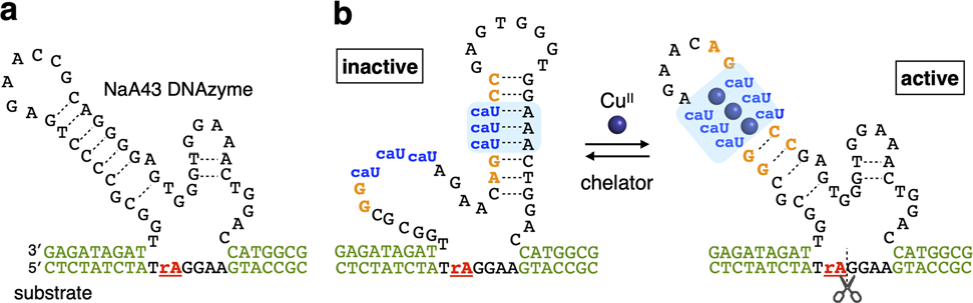
Molecular design of a Cu^II^-responsive allosteric DNAzyme with caU nucleobases (caU-DNAzyme). (a) Sequence of the parent RNA-cleaving DNAzyme (NaA43 DNAzyme). (b) Sequence of caU-DNAzyme. Both the active structure with caU–Cu^II^–caU base pairs and the inactive structure with caU–A base pairs are shown. “rA” in the substrate indicates an adenosine ribonucleotide at the cleavage site.

The Cu^II^-responsive allosteric DNAzyme was logically designed by modifying the base sequence of the known RNA-cleaving NaA43 DNAzyme^11^ (Fig. 2a) in a manner similar to the Gd^III^-responsive DNAzyme with U^OH^ bases.^5^ The NaA43 DNAzyme was chosen because it does not require metal cofactors that can be trapped by common chelators used to selectively remove Cu^II^ ions for reversible regulation of DNAzyme activity (*vide infra*). Since duplex stabilization is more pronounced when three or more consecutive caU–Cu^II^–caU pairs are used,^6^ we decided to incorporate three caU–Cu^II^–caU pairs into the parent NaA43 DNAzyme. Three pairs of caU bases were introduced into the stem region and the surrounding bases (shown in orange) were redesigned to form a different secondary structure in the absence of Cu^II^ ions. The caU-modified DNAzyme (caU-DNAzyme) was expected to undergo a structural change upon addition of Cu^II^ ions, from a catalytically inactive structure with three caU–A base pairs to an active form with three caU–Cu^II^–caU base pairs (Fig. 2b). The plausible secondary structure was simulated using the NUPACK software^12^ (Fig. S1†). The caU bases were replaced with natural T bases to calculate the structure in the absence of Cu^II^, and the potential caU–Cu^II^–caU pairs were changed to G–C base pairs to simulate the structure in the presence of Cu^II^. The results indicated that both the inactive and the active structures can be stably formed *via* the formation of caU–A and caU–Cu^II^–caU base pairs, respectively.

**Fig. 3:**
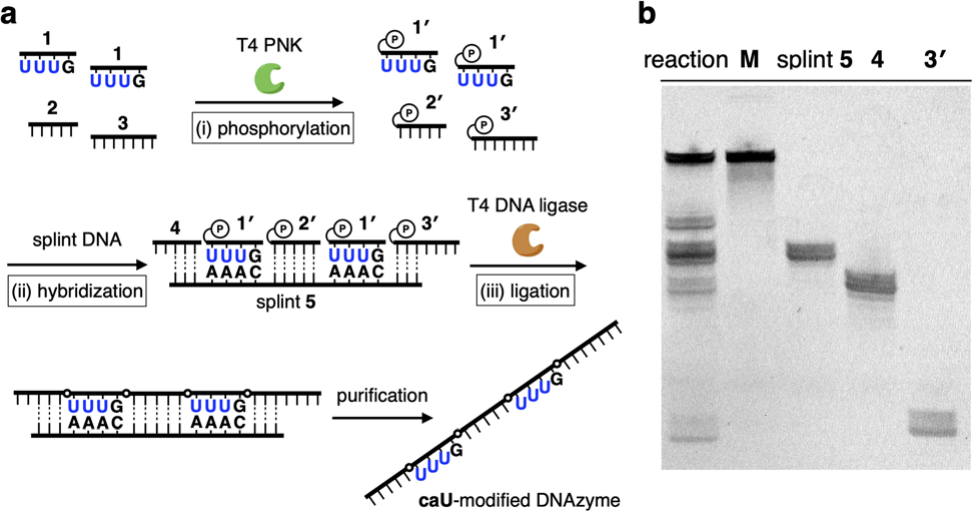
Ligase-mediated synthesis of DNA strands containing caU nucleobases. (a) Reaction scheme. (i) A short DNA strand 1 containing three caU bases (5′-caUcaUcaUG-3′) was phosphorylated by T4 polynucleotide kinase (T4 PNK). (ii) DNA fragments were hybridized with a splint DNA strand 5. (iii) The DNA fragments were ligated by T4 DNA ligase. [1′] = 30 μM, [2′] = [3′] = 12 μM, [4] = [5] = 10 μM, [ligase] = 2 U per μL in quick ligation buffer, 16 °C, 18 h. U in the figure represents a caU base. (b) Denaturing PAGE analysis of the reaction products. The bands were detected after SYBR gold staining. M: chemically synthesized T-DNAzyme, which has T bases instead of caU bases.

A caU-modified DNAzyme strand (58-nt) was synthesized by ligating short DNA fragments (Fig. 3a). The caU-containing strands can be chemically synthesized based on the conventional phosphoramidite chemistry,^6^ but the chemical synthesis requires an additional protecting group on the carboxylate of the caU bases. Although the coupling yield is sufficiently high, incomplete deprotection often makes purification of long oligonucleotides containing multiple caU bases very difficult. Therefore, we expected that the ligase-mediated synthesis would be a suitable strategy to synthesize caU-modified DNAzymes. Since caU nucleobases can form caU–A base pairs with adenine bases on the complementary DNA, caU-modified oligonucleotides were expected to assemble on the splint strand and to be ligated by a standard T4 DNA ligase. A DNA tetramer 5′-caUcaUcaUG-3′ (1) containing three caU nucleotides was used to introduce three consecutive caU bases into the resulting DNA strands. A natural nucleotide G was added at the 3′-terminal so that an additional G–C base pairing would facilitate the hybridization of tetramer 1 to the splint DNA 5. The caU-containing fragment 1 was prepared using an automated DNA synthesizer according to the reported procedure.^6^ Prior to the ligation reaction, fragments 1, 2, and 3 were treated with T4 polynucleotide kinase (T4 PNK) to introduce a phosphate group at the 5′ end (step i). After all the DNA fragments were hybridized to splint 5 (step ii), a ligation reaction was performed using T4 DNA ligase (step iii). Reaction products were analyzed by denaturing polyacrylamide gel electrophoresis (PAGE) (Fig. 3b). After incubation at 16 °C for 18 h, fragment 4 was efficiently consumed and a low mobility band appeared. The mobility of the new band was nearly identical to that of a chemically synthesized T-DNAzyme strand (58-nt) in which the caU nucleotides of the caU-DNAzyme were replaced with natural thymidines (T). Comparing the band intensities, the reaction yield reached 70% or more, confirming that the ligase reaction was proceeding well. The formation of the desired caU-DNAzyme strand was confirmed by MALDI mass spectrometry after isolation ([M–2H]^2−^: calcd 9150.31, found 9150.67, Fig. S2†). It was demonstrated that T4 PNK and T4 DNA ligase successfully phosphorylated and ligated the short strand 1 (5′-caUcaUcaUG-3′) despite the presence of a modified caU base at the 5′ end. Since the desired product can be easily isolated by denaturing PAGE, the ligase-mediated synthesis was shown to be a powerful alternative method to incorporate multiple caU bases into functional DNA sequences.

**Fig. 4:**
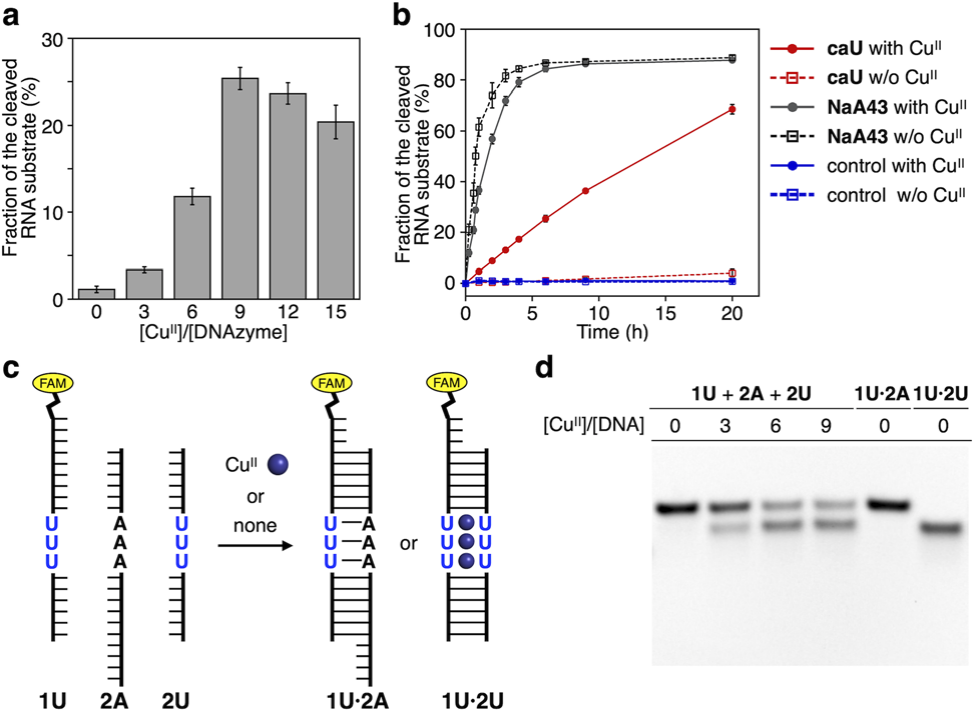
(a) RNA-cleaving activity of caU-DNAzyme in the presence of varying concentrations of Cu^II^ ions. The fractions of the cleaved substrate after a 6 h reaction are shown (see also Fig. S4†). (b) RNA-cleaving activity of caU-DNAzyme and the parent NaA43 DNAzyme in the absence and the presence of 9 equiv. of Cu^II^. T-DNAzyme, in which all caU bases are replaced with natural T bases, was used as a control. [DNAzyme] = 1.0 μM, [substrate] = 10 μM, [CuSO_4_] = 0, 9.0 μM in 10 mM HEPES (pH 7.0), 100 mM NaCl, 25 °C. (c) Cu^II^-mediated change in the hybridization partners of the caU-containing strand (1U). (d) Native PAGE analysis of the hybridization product in the presence of varying amounts of Cu^II^ ions. [DNA] = 2 μM each in 10 mM HEPES (pH 7.0) and 100 mM NaCl. The samples were annealed prior to the analysis. FAM detection.

Using the resulting caU-modified DNAzyme strand, we examined whether the catalytic activity of caU-DNAzyme can be regulated in response to the addition of Cu^II^ ions. DNAzyme-catalyzed RNA-cleaving reactions were performed using 10 equiv. of substrates labeled with a fluorescent dye (FAM). Substrate cleavage was quantitatively evaluated by denaturing PAGE analysis (Fig. S3†). Fig. 4a compares the RNA cleavage reaction catalyzed by caU-DNAzyme in the presence of varying concentrations of Cu^II^ ions. In the absence of Cu^II^ ions, the RNA-cleaving activity of the caU-DNAzyme was greatly suppressed. The DNAzyme activity was found to be enhanced by the addition of Cu^II^ ions; the highest activity was observed when 9 equiv. of Cu^II^ ions were added. Time-course analysis further confirmed the Cu^II^-dependent activation of caU-DNAzyme (Fig. 4b and S4†). Under the same conditions, the catalytic activity of the unmodified NaA43 DNAzyme was reduced by adding Cu^II^ ions (Fig. S5†). A control T-DNAzyme containing natural T bases in place of caU showed no RNA-cleaving activity both in the absence and presence of Cu^II^ ions. These results clearly demonstrate that the addition of Cu^II^ ions enhances the catalytic activity of the caU-modified DNAzyme. In the presence of Cu^II^ ions, the caU-DNAzyme cleaved approximately 70% of the substrate (*i.e.*, 7 equiv.) in 20 h, confirming that the modified DNAzyme maintains multiple turnover ability.

It is most likely that the activity of caU-DNAzyme was switched based on the changes in the base-pairing partners of the caU bases. This was supported by a model experiment using a FAM-labeled strand containing three caU bases in the middle (1U) (Fig. 4c and S6†). The DNA 1U was annealed with two complementary strands 2U and 2A containing three caU or A bases, respectively, and the hybridization products were analyzed by native PAGE (Fig. 4d). Under Cu^II^-free conditions, only the duplex 1U·2A with caU–A pairs was formed. In the presence of Cu^II^ ions, the duplex 1U·2U with caU–Cu^II^–caU pairs was formed (up to about 60%). These results clearly show the Cu^II^-mediated change in the hybridization partners, which is the driving force behind the allosteric regulation of the caU-DNAzyme.

The catalytically active form contains three caU–Cu^II^–caU base pairs, but the maximum activity was observed in the presence of 9 equiv. of Cu^II^ ions (Fig. 4a). This inconsistency can be explained by the stability of the caU–A base pairs in addition to the caU–Cu^II^–caU pairs.^6^ Melting experiments (Fig. S7†) showed that the model 15-bp duplex 1U′·2U, containing three caU–caU pairs, exhibited the highest melting temperature (*T*_m_) in the presence of 3 equiv. of Cu^II^ ions, due to the quantitative formation of caU–Cu^II^–caU pairs. On the other hand, the stability of duplex 1U′·2A′ with three caU–A pairs decreases with increasing amounts of Cu^II^ ions, possibly due to the binding of Cu^II^ ions to the caU bases.^6^ The difference in the *T*_m_ values of duplexes 1U′·2U and 1U′·2A′ was maximal when more than 3 equiv. of Cu^II^ were added. In fact, the hybridization experiments (Fig. 4d) showed that an excess of Cu^II^ ions are required to change the hybridization partners of the caU bases. Therefore, the requirement for an excess of Cu^II^ ions to activate the caU-DNAzyme suggests that the DNAzyme functions through both Cu^II^-mediated destabilization of the caU–A pairs (inactive state) and Cu^II^-mediated caU–Cu^II^–caU base pair formation (active state) exactly as designed.

The apparent first-order rate constants (*k*_obs_) for the DNAzyme reactions were estimated from the initial rates (Table 1). The catalytic activity of caU-DNAzyme was found to increase by approximately 21-fold with the addition of 9 equiv. of Cu^II^ ions. The maximum activity of the caU-DNAzyme (*k*_obs_ = 4.2 × 10^−2^ h^−1^) was lower than that of the unmodified NaA43 DNAzyme (*k*_obs_ = 4.7 × 10^−1^ h^−1^). This may be due not only to the incomplete transformation into the active state, but also to the structural distortion caused by the caU–Cu^II^–caU base pairs.^6^ As indicated by circular dichroism (CD) analysis in the previous study,^6^caU–Cu^II^–caU base pairing can unwind the stem duplex to some extent. Introducing the caU base at a position more distant from the catalytic core would reduce the negative effect on the DNAzyme activity. It is noteworthy that the Cu^II^-mediated activation of caU-DNAzyme (21-fold) was much more efficient than that of a Cu^II^-responsive H-modified DNAzyme (5.9-fold), which was developed by incorporating an H–Cu^II^–H base pair into the same NaA43 DNAzyme. The results clearly show that the bifacial caU nucleobases, which form both hydrogen-bonded caU–A and metal-mediated caU–Cu^II^–caU base pairs, are useful for metal-responsive switching of DNA functions.

**Table 1:** Apparent first-order rate constants (*k*_obs_) for the DNAzyme-catalyzed RNA cleavage reactions

DNAzyme	*k*_obs_/h^−1^		Ratio
	Cu^II^+	Cu^II^–	
caU-DNAzyme	4.3 × 10^−2^ b	2.0 × 10^−3^	21
NaA43 DNAzyme	4.7 × 10^−1^ b	8.5 × 10^−1^	0.56
T-DNAzyme	<1.0 × 10^−3^ b	<1.0 × 10^−3^	—
H-modified DNAzyme^a^	2.8 × 10^−1^ c	4.7 × 10^−2^	5.9

a A NaA43 DNAzyme modified with a pair of hydroxypyridone (H) nucleobases.^9*b*^

b In the presence of 9 equiv. of Cu^II^ ions.

c In the presence of 1 equiv. of Cu^II^ ions.

We further carried out the DNAzyme reactions in the presence of Hg^II^ ions that can mediate caU–Hg^II^–caU base pairing^6^ (Fig. S8†). In contrast to Cu^II^ ions, the addition of Hg^II^ ions did not activate caU-DNAzyme at all. Note that the activity of the unmodified NaA43 DNAzyme was significantly reduced by the addition of Hg^II^ ions. This is probably due to the undesired binding of Hg^II^ ions to the natural T bases. These results clearly demonstrate the advantage of using metal ions that do not interact strongly with natural bases (*e.g.*, Cu^II^), especially with long oligonucleotides such as DNAzymes.

**Fig. 5:**
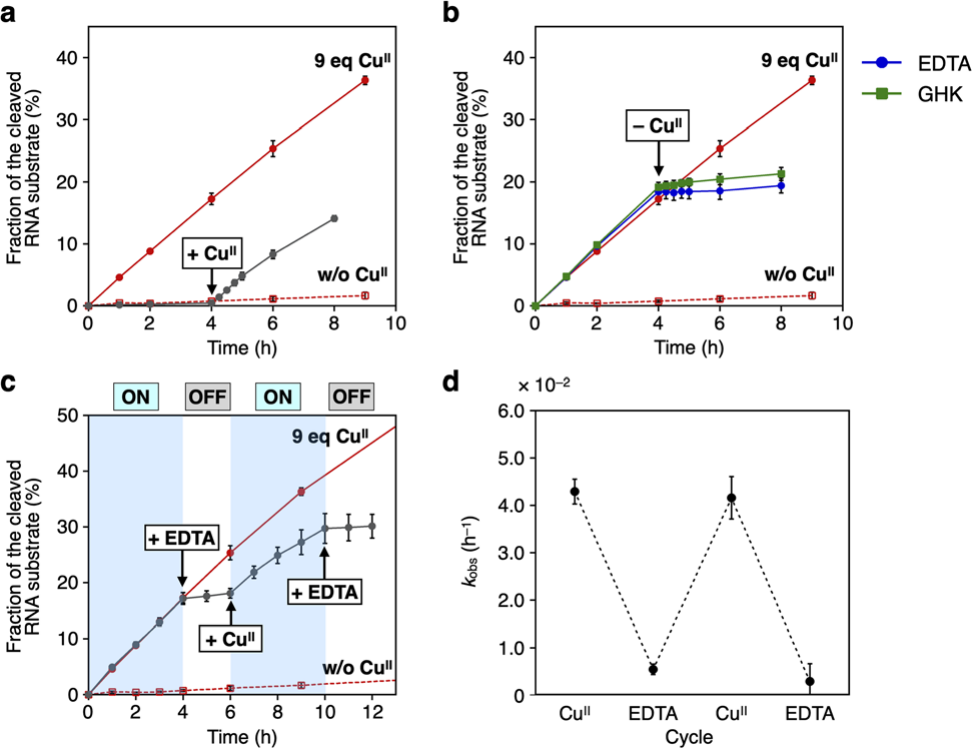
Cu^II^-dependent regulation of the RNA-cleaving activity of caU-DNAzyme. (a) Activation of caU-DNAzyme by the addition of Cu^II^ ions (9 equiv.). (b) Deactivation of caU-DNAzyme by the removal of Cu^II^ ions with a chelating agent EDTA or a Cu^II^-binding tripeptide (GHK) (9 equiv.). (c) Iterative switching of the DNAzyme activity. Cu^II^ (9 equiv.) and EDTA (9 equiv.) were alternately added. [DNAzyme] = 1.0 μM, [substrate] = 10 μM, 25 °C. The activities of caU-DNAzyme in the absence (red dotted lines) and presence of Cu^II^ ions (red solid lines) are also shown. (d) Apparent first-order rate constant (*k*_obs_) for each step. *N* = 3. Error bars indicate standard errors.

The RNA-cleaving activity of caU-DNAzyme was reversibly regulated in response to Cu^II^ ions during the reaction (Fig. 5). The reaction was initiated in the absence of Cu^II^ ions, and after 4 h, Cu^II^ ions (9 equiv.) were added. The reaction rate was immediately increased to *k*_obs_ = 3.9 × 10^−2^ h^−1^, which is comparable to the rate observed when the reaction was initiated with Cu^II^ ions (Fig. 5a). In a similar manner, removal of Cu^II^ ions was shown to inactivate caU-DNAzyme. Addition of the chelating agent EDTA or Cu^II^-binding peptide (GHK)^13^ in equimolar amounts with Cu^II^ ions (9 equiv.) immediately slowed the reaction (*k*_obs_ = 2.5 × 10^−3^ h^−1^ and 5.3 × 10^−3^ h^−1^, respectively) (Fig. 5b). The addition of sodium ascorbate, which can reduce Cu^II^ to Cu^I^, also decreased the activity of caU-DNAzyme to *k*_obs_ = 3.6 × 10^−3^ h^−1^ (Fig. S9†). These results demonstrate that the activity of caU-DNAzyme can be rapidly switched by the addition, removal, and reduction of Cu^II^ ions under isothermal conditions. Alternate addition of Cu^II^ ions (9 equiv.) and EDTA (9 equiv.) cycled the on–off switching of caU-DNAzyme (Fig. 5c). The *k*_obs_ values in each step demonstrate a clear switching in the caU-DNAzyme activity in response to Cu^II^ (Fig. 5d).

## Conclusions

In summary, a Cu^II^-responsive allosteric DNAzyme was developed by introducing 5-carboxyuracil (caU) nucleobases into a known DNAzyme sequence. The caU-modified DNAzyme (caU-DNAzyme) was enzymatically synthesized by joining short caU-containing fragments with a standard T4 DNA ligase. The ligase-mediated synthesis was possible because the caU base was structurally similar to the natural T base and could form a Watson–Crick-like base pair with the A base on the splint DNA. The base sequence of caU-DNAzyme is logically designed to form both the catalytically inactive structure by caU–A base pairing and the active form by metal-mediated caU–Cu^II^–caU base pairing. The activity of caU-DNAzyme was enhanced 21-fold by the addition of Cu^II^ ions and could be turned on and off during the reaction by the addition and removal (or reduction) of Cu^II^ ions. These results demonstrate that the caU-modified DNAzyme was allosterically regulated through metal-mediated base-pair switching between caU–A and caU–Cu^II^–caU. The use of Cu^II^ is essential to induce base-pair switching of the caU base. The caU base can form other types of metal-mediated base pairs such as caU–Hg^II^–T, caU–Ag^I^–C, and caU–Cu^II^–G.^6^ Therefore, caU-modified DNAs are expected to be further applied in constructing more complex DNA systems responsive to multiple metal ions.

This study confirms that metal-responsive DNA systems can be logically designed based on metal-mediated base-pair switching of bifacial caU nucleobases. The strategic design of caU-modified DNAzymes is expected to be applied to other types of DNAzymes^14^ and other functional DNAs as well. Ligase-mediated synthesis provides a simple way to incorporate caU bases into longer DNA sequences and is advantageous for sequence screening. Thus, it is suggested that the incorporation of bifacial caU bases is a powerful strategy for creating a variety of Cu^II^-responsive DNA molecular systems. The range of metal ions used could be expanded by developing other types of bifacial nucleobases with a different metal coordinating functionality at the 5-position of pyrimidine bases. In fact, Gd^III^-responsive DNA systems have been developed using cognate U^OH^ nucleobases.^5^ The bifacial 5-modified pyrimidine bases are expected to be introduced into DNA *via* the ligase-mediated synthesis, similar to the case with caU. Therefore, metal-mediated base-pair switching of bifacial nucleobases and the ligase-mediated synthesis have the potential to be versatile tools for building DNA-based stimuli-responsive systems such as biosensors, molecular machines, and computing devices. Further applications of bifacial caU nucleobases to metal-triggered operation of DNA nanoarchitectures and DNA logic circuits are currently under investigation.

## Data availability

All the data supporting this study are included in the main text and the ESI.†

## Author contributions

Y. T. and M. S. conceived and directed the study. H. Z. and K. M. performed the experiments and analyzed the data with the aid of Y. T. and L. H. All the authors prepared the manuscript.

## Conflicts of interest

There are no conflicts to declare.

## Supplementary Material

SC-015-D3SC05042D-s001
