# Supplementary material for: Ligase-mediated synthesis of CuII-responsive allosteric DNAzyme with bifacial 5-carboxyuracil nucleobases
Source: Chem Sci. 2024 Jan 20;15(7):2365–70. doi: 10.1039/d3sc05042d (PMC10866359; doi:10.1039/d3sc05042d)
Supplement: SC-015-D3SC05042D-s001 [file SC-015-D3SC05042D-s001.pdf]

Electronic Supplementary Material (ESI) for Chemical Science.
This journal is © The Royal Society of Chemistry 2024

Electronic Supplementary Information

Ligase-mediated Synthesis of Cu^{II}-responsive Allosteric DNAzyme with Bifacial 5-Carboxyuracil Nucleobases

Yusuke Takezawa,* Hanci Zhang, Keita Mori, Lingyun Hu and Mitsuhiko Shionoya*

Department of Chemistry, Graduate School of Science, The University of Tokyo, 7-3-1 Hongo, Bunkyo-ku, Tokyo 113-0033, Japan.

E-mail: takezawa@chem.s.u-tokyo.ac.jp; shionoya@chem.s.u- tokyo.ac.jp

Table of Contents

1. **Experimental methods**
2. **Supporting tables and figures**
3. **References**

1. Experimental methods

Materials and equipment. All the natural DNA strands, including 6-carboxyfluorescein (FAM)-labeled substrates containing a riboadenosine (rA), were purchased from Japan Bio Service Co., Ltd. (Saitama, Japan) at HPLC purification grade. The DNA tetramer containing 5-carboxyuracil nucleotides (5′-**caUcaUcaU**G-3′, **1**) was chemically synthesized on an automated DNA synthesizer (NTS M-4-MX DNA/RNA synthesizer) according to the reported procedure[1] and purified by reverse phase HPLC (Waters XBridge C18 column, 0.1 M TEAA (pH 7.0)/MeCN gradient, 60 °C). The tetranucleotide **1** was identified by ESI-TOF mass spectrometry (Waters Micromass LCT premier) (*m*/*z* calcd for $[C_{40}H_{47}N_{11}O_{31}P_3–H]^-$:1268.15; found: 1268.23). The **caU**-containing DNA strands used for the duplex melting analysis (**1U′** and **2U**) were synthesized in the previous study.[1] The FAM-labeled **caU**-containing strand used for the hybridization experiment (**1U**) was synthesized by ligating **1U′** and a FAM-labeled pentamer (5′-FAM-ACCTC-3′) using T4 DNA ligase and characterized by MALDI-TOF mass spectrometry (Bruker Autoflex III) (*m*/*z* calcd for $[C_{222}H_{272}N_{64}O_{138}P_{20}–H]^-$: 6660.09; found: 6658.08). The concentration of DNA strands was determined based on the UV absorbance at 260 nm. The molar extinction coefficients (ε_{260}) of the DNA strands were calculated by the nearest neighbor method, in which ε_{260} of **caU** nucleotide was assumed to be 7.7 × 10^3 $M^{-1}cm^{-1}$).[1] $CuSO_4·5H_2O$ (99.5% purity) and Glycyl-L-histidyl-L-lysine (GHK) were purchased from FUJIFILM Wako Pure Chemical Industries and Arc Pharm, Inc., respectively. Denaturing polyacrylamide gel electrophoresis (PAGE) was carried out with 20% polyacrylamide gel containing 7 M urea. The gels were imaged and analyzed by using Gel Doc EZ Imager and Image Lab software (Bio-Rad).

Enzymatic synthesis of caU-modified DNAzyme. Three short DNA fragments (**1**, **2**, and **3**) were phosphorylated by using T4 polynucleotide kinase (T4 PNK) to give 5′-phosphorylated strands (**1′**, **2′**, and **3′**). The DNA strands were dissolved in an NEB Quick Ligation buffer (66 mM Tris–HCl (pH 7.6), 10 mM $MgCl_2$, 1 mM DTT, 1 mM rATP, 7.5% PEG6000). After the addition of T4 PNK, the mixture was incubated at 37 °C for 1 h. The final concentration of each component was as follows: [Short DNA fragments] = 100 μM each, [T4 PNK] = 0.1 U μL^{-1}. The reaction solution was then heated at 95 °C for 5 min to inactivate the T4 PNK. Subsequently, a DNA fragment **4** and a splint strand **5** was added to the resultant solution and the mixture was annealed prior to the reaction (85 °C → 4 °C, 1.0 °C min^{-1}). After T4 DNA ligase was added, the mixture was incubated at 16 °C for 18–20 h. The final concentration of each component was as follows: [**1′**] = 30 μM, [**2′**] = [**3′**] = 12 μM, [**4**] = [**5**] = 10 μM, [T4 DNA ligase] = 2 U μL^{-1}. The

reaction was stopped by the addition of a 3:1 mixture of 7 M urea and a loading solution (30% glycerol, 0.25% bromophenol blue), and the mixture was immediately heated at 95 °C. The products were analyzed by denaturing PAGE with SYBR Gold staining. The product was purified by denaturing PAGE, ultrafiltration (10 kDa MWCO), and isopropanol precipitation. The **caU**-DNAzyme strand was characterized by MALDI-TOF mass spectrometry (Bruker Autoflex III) using a mixture of 3-hydroxypicolinic acid (3-HPA) and ammonium citrate as a matrix.

RNA-cleaving reaction by DNAzymes. DNAzyme strands were annealed (85 °C → 25 °C, 1.0 °C min^{-1}) in 10 mM HEPES buffer (pH 7.0) containing 100 mM NaCl in the absence or in the presence of $CuSO_4$. A FAM-labeled substrate strand was then added to initiate the RNA-cleaving reaction. The final concentration of each component was as follows: [DNAzyme] = 1.0 μM, [substrate] = 10 μM, [$CuSO_4$] = 0 or 9 μM (otherwise noted). After the incubation at 25 °C, an aliquot of the sample was taken at the designated time points. The reaction was stopped by the addition of a 3:1 mixture of 7 M urea and a loading solution (30% glycerol, 0.25% bromophenol blue), and the mixtures were stored at –28 °C until the time course was completed. The cleavage of the substrate was analyzed by denaturing PAGE. The fractions of the cleaved substrate (F) were calculated as follows:

F (%) = I_c / (I_c + I_u) × 100,

where I_c and I_u are the band intensities of the cleaved product and the uncleaved substrate, respectively. The apparent first-order rate constants (k_{obs}) were calculated from the initial rates, which were determined from the time points when F was less than 20%.

DNAzyme reactions in the presence of Hg^{II} ions. DNAzyme strands were annealed (85 °C → 25 °C, 1.0 °C min^{-1}) in a reaction buffer (10 mM HEPES buffer (pH 7.0), 100 mM $NaNO_3$) in the absence or in the presence of $Hg(ClO_4)_2$. Note that NaCl was replaced with $NaNO_3$ in the reaction buffer to prevent the interaction between Hg^{II} and Cl^- ions. The following procedures are the same as that in the presence of Cu^{II}. The final concentration of each component was as follows: [DNAzyme] = 1.0 μM, [substrate] = 10 μM, [$Hg(ClO_4)_2$] = 0, 3, and 6 μM.

Regulation of DNAzyme activity during the reaction. For the DNAzyme activation by the addition of Cu^{II} ions (Fig. 5a), the RNA-cleaving reaction was started without Cu^{II} ions and 1.0 equiv of $CuSO_4$ (1.0 equiv) was added after 4 h. For the deactivation by the removal of Cu^{II} ions (Fig. 5b), the reaction was started in the presence of Cu^{II} ions (1.0 equiv). After 4-h reaction, EDTA (9 equiv) or a Cu^{II}-binding tripeptide GHK (9 equiv) was added to remove Cu^{II} ions. For

the inactivation by the reduction of Cu^{II} ions (Fig. S8), sodium ascorbate (90 equiv) was added instead. For iterative switching of the DNAzyme activity (Fig. 5c), $CuSO_4$ (9 equiv) and EDTA (9 equiv) were alternately added at the defined time points. The reaction progress was monitored by denaturing PAGE as described above.

Hybridization experiments. The samples were prepared by mixing a FAM-labeled **caU**-containing strand (**1U**), a complementary strand containing **caU** bases (**2U**), and a complementary strand containing A bases (**2A**) (2.0 µM each) in 10 mM HEPES buffer (pH 7.0) containing 100 mM NaCl. The mixture was annealed (85 °C → 4 °C, 1.0 °C min^{-1}) in the presence of varying concentrations of Cu^{II} ions. Native PAGE was performed with 20% polyacrylamide gel in a cool incubator (4 °C). Since the lengths of **2U** and **2A** are different, the hybridization products **1U·2A** and **1U·2U** can be separated in the gel. The bands were detected by FAM fluorescence and the yield of each product was calculated by comparing the intensities of the two bands observed in the same lane.

Duplex melting analysis. The DNA strands were annealed (85 °C → 4 °C, 1.0 °C min^{-1}) in 10 mM HEPES buffer (pH 7.0) containing 100 mM NaCl in the presence of Cu^{II} ions. Absorbance at 260 nm (A_{260}) was recorded on UV-1700 or UV-1800 spectrophotometer (Shimadzu) equipped with a TMSPC-8 temperature controller while the temperature was raised from 5 °C to 85 °C at the rate of 0.2 °C min^{-1}. The melting temperatures (T_m) were determined as an inflection point of a melting curve using a T_m analysis software LabSolutions (Shimadzu) with a 17-point adaptive smoothing program.

2. Supporting tables and figures

Table S1: Sequences of DNA strands used in this study.

DNA strands	Sequence
**1**	5′-**caUcaUcaU** G-3′
**2**	5′-ACA AGA-3′
**3**	5′-GCG CGG TTA GAT AGA G-3′
**4**	5′-GCG GTA CCA GGT CAA AGG TGG GTG AGC C-3′
**5**	5′-CTA ACC GCG CCA AAT CTT GTC AAA GGC TCA CCC A-3′
**caU**-DNAzyme	5′-GCG GTA CCA GGT CAA AGG TGG GTG AGC C**caUcaU caU**GA CAA GA**caU caUcaU**G GCG CGG TTA GAT AGA G -3′
NaA43 DNAzyme	5′-GCG GTA CCA GGT CAA AGG TGG GTG AGG GGA CGC CAA GAG TCC CCG CGG TTA GAT AGA G-3′
**T**-DNAzyme	5′-GCG GTA CCA GGT CAA AGG TGG GTG AGC CTT TGA CAA GAT TTG GCG CGG TTA GAT AGA G-3′
substrate	5′-FAM-CTC TAT CTA T(rA)G GAA GTA CCG C-3′
**1U**	5′-FAM-ACC TCC ACA TT**caU caUcaU**G TTG TA-3′
**2U**	3′-GTG TAA **caUcaUcaU** CAA CAT-5′
**2A**	3′-GTG TAA **AAA** CAA CAT AGT CGT-5′
**1U′**	5′-CAC ATT **caUcaUcaU** GTT GTA-3′
**2A′**	3′-GTG TAA **AAA** CAA CAT-5′

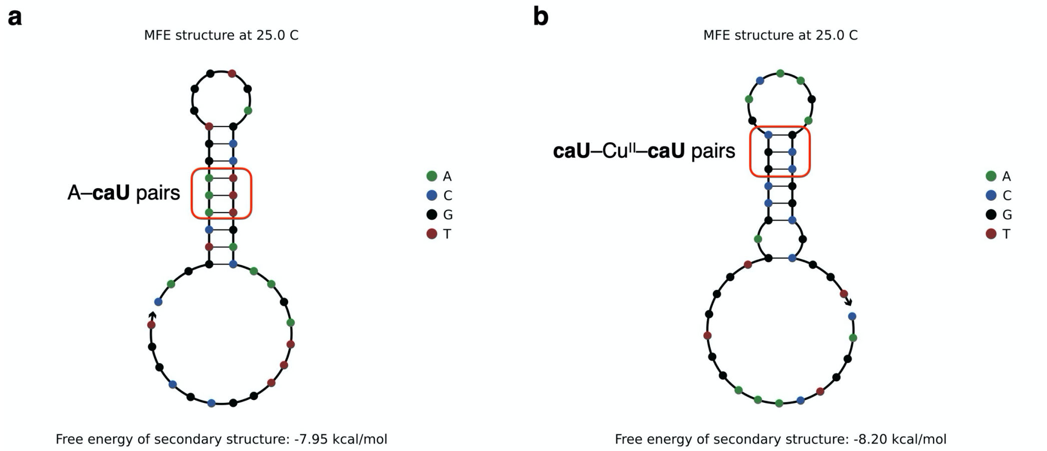

Fig. S1. Secondary structures of **caU**-DNAzyme simulated by NUPACK software. (a) Structure in the absence of Cu^{II} ions. All **caU** bases were replaced with T bases. (b) Structure in the presence of Cu^{II} ions. Potential **caU**–Cu^{II}–**caU** base pairs were replaced with G–C pairs. The substrate binding domain was removed for sake of simplicity.

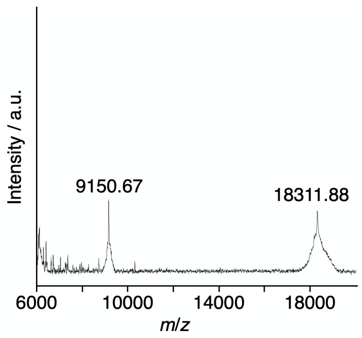

Fig. S2. MALDI-TOF mass spectrum of the isolated **caU**-DNAzyme strand. Negative mode. $[M – 2H]^{2–}$: calcd 9150.31, found 9150.67.

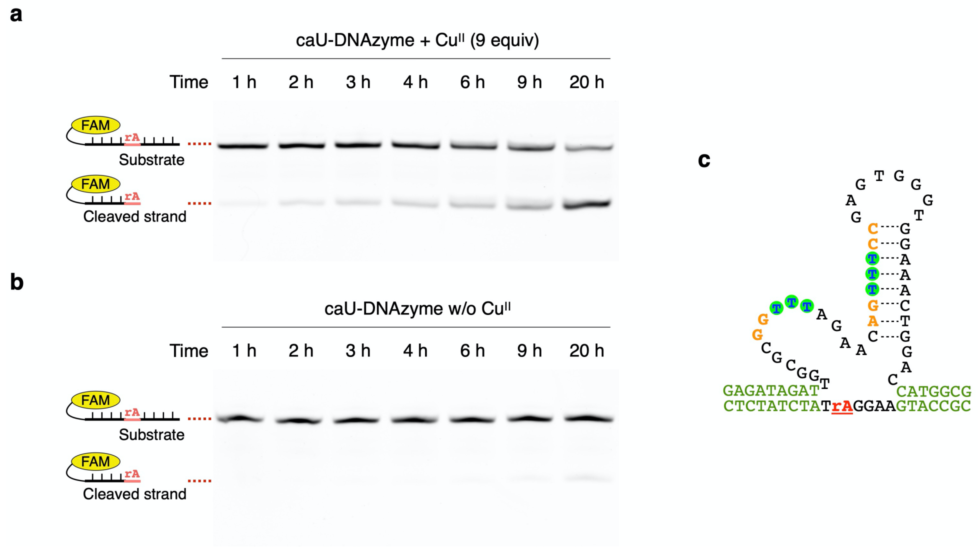

Fig. S3. Denaturing PAGE analysis of the RNA-cleaving reactions catalyzed by **caU**-DNAzyme in the presence (a) and absence (b) of Cu^{II} ions (9 equiv). [**caU**-DNAzyme] = 1.0 µM, [substrate] = 10 µM, $[CuSO_4]$ = 0 or 9.0 µM in 10 mM HEPES (pH 7.0), 100 mM NaCl, 25 °C. FAM detection. (c) Base sequence of T-DNAzyme used as a control (Fig. 4b). The **caU** bases in **caU**-DNAzyme are replaced with natural T bases.

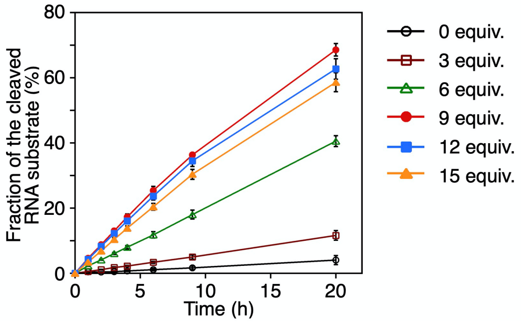

Fig. S4. RNA-cleaving activity of **caU**-DNAzyme in the presence of varying concentrations of Cu^{II} ions. [**caU**-DNAzyme] = 1.0 μM, [substrate] = 10 μM, [$CuSO_4$]/[**caU**-DNAzyme] = 0, 3, 6, 9, 12, and 15 in 10 mM HEPES (pH 7.0), 100 mM NaCl, 25 °C. $N = 3$. Error bars indicate standard errors.

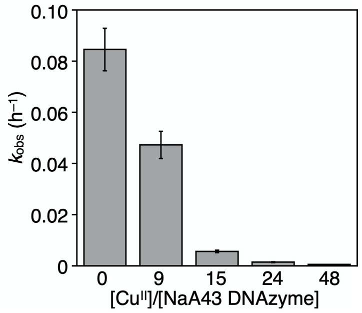

Fig. S5. Apparent first-order rate constants (k_{obs}) for the RNA-cleaving reaction catalyzed by the parent NaA43 DNAzyme in the presence of Cu^{II} ions. [NaA43 DNAzyme] = 1.0 μM, [substrate] = 10 μM, [$CuSO_4$]/[NaA43 DNAzyme] = 0, 9, 15, 24, and 48 in 10 mM HEPES (pH 7.0), 100 mM NaCl, 25 °C. N = 3. Error bars indicate standard errors.

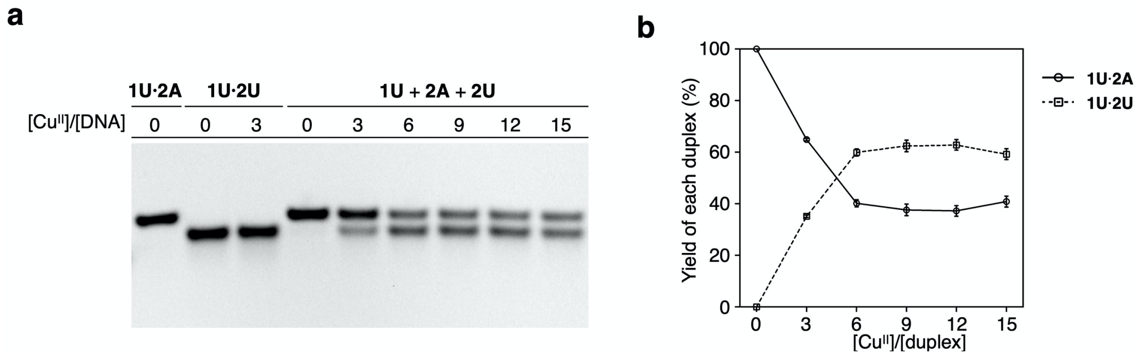

Fig. S6. Cu^{II}-mediated alteration of the hybridization partner of **caU**-containing strand **1U**. (a) Native PAGE analysis of the hybridization product in the presence of varying amounts of Cu^{II} ions. [DNA] = 2 μM each, [$CuSO_4$]/[DNA] = 0, 3, 6, 9, 12, and 15, in 10 mM HEPES (pH 7.0), 100 mM NaCl. The reaction scheme is shown in Fig. 4c. The samples were annealed prior to the analysis. FAM detection. (b) Yields of duplex **1U**·**2U** containing **caU**–Cu^{II}–**caU** base pairs and duplex **1U**·**2A** containing **caU**–A base pairs.

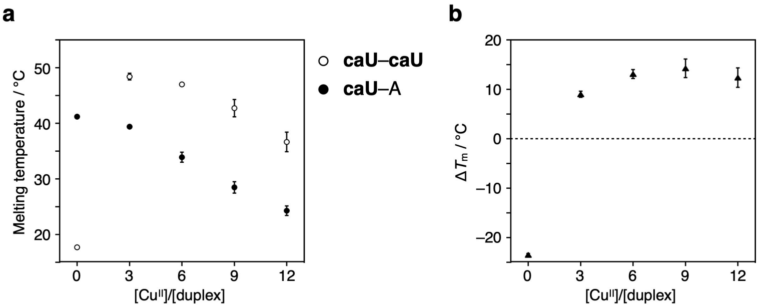

Fig. S7. (a) Melting temperatures (T_m) of a 15-bp DNA duplex **1U′·2U** containing three **caU**–**caU** base pairs (white circle) and a duplex **1U′·2A′** with three **caU**–A base pairs (black circle) in the presence of varying amounts of Cu^{II} ions. [duplex] = 2.0 μM, [$CuSO_4$]/[duplex] = 0, 3, 6, 9, and 12 in 10 mM HEPES (pH 7.0), 100 mM NaCl, 0.2 °C min^{-1}. All the samples were annealed before the measurements. (b) Differences between the melting temperatures (T_m) of duplex **1U′·2U** and those of **1U′·2A′** in the presence of varying amounts of Cu^{II} ions. $\Delta T_m = T_m$(**1U′·2U**) – T_m(**1U′·2A′**). N = 3. Error bars indicate standard errors.

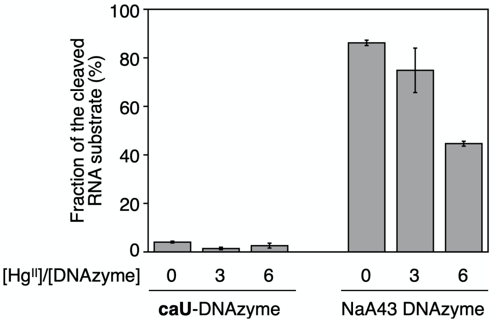

Fig. S8. RNA-cleaving activity of **caU**-DNAzyme and NaA43 DNAzyme in the presence of various concentrations of Hg^{II} ions. The fractions of cleaved substrate after 6-h reaction are shown. [DNAzyme] = 1.0 μM, [substrate] = 10 μM, [$Hg(ClO_4)_2$]/[DNAzyme] = 0, 3, and 6 in 10 mM HEPES (pH 7.0), 100 mM $NaNO_3$, 25 °C, 6 h $N \geq 2$. Error bars indicate standard errors.

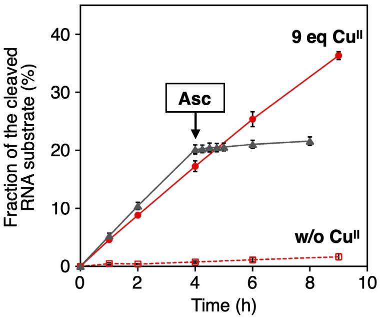

Fig. S9. Deactivation of **caU**-DNAzyme by the reduction of Cu^{II} ions with sodium ascorbate (Asc) (90 equiv). The reaction was started with 9 equiv of Cu^{II} ions. At 4 h, 90 equiv of Asc were added. [DNAzyme] = 1.0 μM, [substrate] = 10 μM, 25 °C. N = 3. Error bars indicate standard errors. The activities of **caU**-DNAzyme in the absence (red dotted lines) and presence of Cu^{II} ions (red solid lines) are also shown.

3. References

[1] Y. Takezawa, A. Suzuki, M. Nakaya, K. Nishiyama and M. Shionoya, *J. Am. Chem. Soc.*, 2020, **142**, 21640–21644.
